# Evaluation of Allelopathic Activity of Chinese Medicinal Plants and Identification of Shikimic Acid as an Allelochemical from *Illicium verum* Hook. f.

**DOI:** 10.3390/plants9060684

**Published:** 2020-05-28

**Authors:** Aniya, Yoshihiro Nomura, Fuerdeng, Kwame Sarpong Appiah, Yoshiharu Fujii

**Affiliations:** 1 Department of Applied Life Science, United Graduate School of Agriculture, Tokyo University of Agriculture and Technology, Fuchu Tokyo 183-8509, Japan; s178822w@st.go.tuat.ac.jp; 2 Department of Sustainable Production, Institute of Environmental Protection, Urumqi 830000, Xinjiang, China; fabiao@vip.126.com; 3 Department of Biological Production Science, United Graduate School of Agriculture, Tokyo University of Agriculture and Technology, Fuchu, Tokyo 183-8509, Japan; s190222w@st.go.tuat.ac.jp (K.S.A.); yfujii@cc.tuat.ac.jp (Y.F.)

**Keywords:** allelopathy, sandwich method, *Illicium verum* Hook. f., phytotoxicity, weed management, sustainable agriculture

## Abstract

This study focused on the potential allelopathy of 50 species of Chinese medicinal plants, which are mainly distributed in the Xinjiang Uyghur Autonomous Region, Inner Mongolia, and Yunnan Province. The “sandwich method” was adopted and used for the screening for allelopathic potential among these plant species. Further phytotoxic evaluation of the candidate species was conducted by applying plant extracts to crops and weed species. The results of this study indicated that among the 50 medicinal plant species evaluated, the fruits of *Illicium verum* Hook. f. (star anise) showed the most significant allelopathic potential through the leaf leachates. Shikimic acid was identified to be the main bioactive compound (about 7% dry weight) in star anise by reversed-phase High Performance Liquid Chromatography (RP-HPLC) analysis. The phytotoxic bioassay indicated that both the crude extract of the Chinese star anise and the synthetic shikimic acid showed strong inhibitory activity on the radicle and hypocotyl growth of lettuce. The radicle growth inhibition of lettuce caused by the crude extract of star anise could be explained by the contribution of the biological activity of shikimic acid. In conclusion, shikimic acid could be a putative allelochemical in the fruits of *Illicium verum* and could be utilized in sustainable weed management.

## 1. Introduction

The management of weeds on the field is an important measure for crop production in most countries including China. Weeds constantly compete with crop plants, causing considerable crop productivity losses in many agricultural systems. In effect, weeds have been documented as serious plant pests since ancient times [1]. Weeds have always played a significant role throughout the domestication of crop plants, necessitating the practice of weed control measures [1]. In modern agricultural practices, herbicides are widely used to control weeds, but there are some environmental concerns about their excessive use. Furthermore, cases of herbicide resistant/tolerant weeds are gradually on the rise [2]. Allelopathy is a phenomenon observed in many organisms (especially in plants) that involves the production and release of bioactive compounds into the environment. These compounds (allelochemicals) are released from the aerial or underground parts in the form of root exudation, leachates by dews and rains, volatilization, or decomposition of plant tissue. These bioactive compounds influence the growth and development (inhibitory or stimulatory) of neighboring plants [3]. The weed suppressive and antimicrobial activities of some allelopathic plants or their extracts have been reported [4]. There are increasing opportunities to utilize allelopathic species or their extracts to minimize the use of synthetic herbicides in sustainable agriculture [5]. From an ecological perspective, allelopathy may play an important role in the process of biological invasion. Some invasive plant species are perceived to be successful because they possess novel bioactive compounds that function as allelopathic agents or as mediators of the new plant–plant interactions [6]. Some effects of allelochemicals on the growth and development of susceptible plants include, but are not limited to, reduced radicle and shoot extension, darkened and/or swollen seeds, curling of root axis, discoloration of seeds, lack of root hairs, necrosis, increased number of seminal roots, and reduced dry weight accumulation [7]. Previous studies on the screening for potential allelopathic species by water extraction method found that medicinal plants showed relatively strong allelopathic activity [8,9]. Medicinal plants are an important part of the biodiversity of China, and there are about 10,608 medicinal higher plant species. This accounts for about 83.4% of all medicinal biological resources in China [10] and about 30.3% of all medicinal plants in the world [11]. However, studies about these Chinese medicinal plant species for allelopathic potential have not been widely reported. In this study, we aimed to evaluate the potential allelopathic activity of Chinese medicinal plants and identify the allelochemical responsible for the plant growth inhibitory effects of the candidate species.

## 2. Materials and Methods

### 2.1. Plant Materials

In this study, the leaf leachates of 50 Chinese medicinal plant species were obtained from the Xinjiang Institute of Ecology and Geography and the Beijing Hospital of Traditional Chinese Medicine to evaluate their allelopathic potentials. Among the collected plant samples, 18 plant species are distributed in Yunnan Province, 17 plant species are distributed in Xinjiang Uyghur Autonomous Region, and 15 species are distributed in Guizhou Province, Inner Mongolia, and Tibetan Autonomous Region. All the plant samples were dried in a hot air circulation oven at 60ºC for approximately 4 h and were ground into powder. Finally, they were put into paper bags for further use. Seeds of *Lactuca sativa* L., *Trifolium pratense* L., *Trifolium repens* L., *Medicago sativa* L., and *Lotus corniculatus* L. (Takii Seed Co., Ltd., Kyoto, Japan) were used as receptor plants in the test for plant growth inhibitory activities among the collected samples and the candidate species.

### 2.2. Screening of Plant Species for Potential Plant Growth Inhibition by Sandwich Method

Leaf leachate is one of the major routes of allelochemical release into the environment and, just like other routes of allelochemical release, requires a specific bioassay to evaluate plants with such potential. The sandwich method was developed to evaluate the allelopathic activity of plants through leaf leachates under controlled laboratory conditions [12]. It is a less time-consuming bioassay method that can be used to screen a large number of samples [12,13,14]. In this study, either 10 or 50 mg of dried sample was placed into each well of a six-well multi-dish plastic plate. Agar growth medium in the sandwich method was found to be best for the lettuce seedling growth in a previous study [14]. Therefore, we used agar (Nakalai Tesque, Ltd., Kyoto, Japan; 0.75% *w/v*) as the growth medium after sterilization (115 °C, for 2 h). Lettuce (*Lactuca sativa* L. Great Lakes 366, Takii Seed Co. Ltd., Japan) was used as the test plant material in the initial bioassay because of its uniformity in germination and sensitivity to allelochemicals [14]. After placing five seeds of lettuce on the agar medium, the multi-dish plastic plates were sealed with plastic tape and covered with aluminum foil for incubation under completely dark conditions for 72 h at 22 °C. The blank control was set up without any plant material, and five lettuce seeds were placed on the agar medium in each well. The lettuce radicle and hypocotyl lengths were measured and compared with that of the control to calculate elongation percentage (Equation (1)). Each experiment was repeated three times, and the average value was obtained.

*Elongation* (%) = x/y × 100% (1)

where x = average of treatment radicle/hypocotyl length and y = average of control radicle/hypocotyl length

For the evaluation of the allelopathic activity, the concept of the “standard deviation variance” (SDV) was adopted [8,15]. For the statistical analysis, the mean and standard deviation were calculated and the criterion of the SDV was evaluated by Microsoft Excel 2010. The severity of inhibition on growth elongation was defined at 3 levels: from the least to the highest effect, respectively. Level one (*) = M—0.5 × SD, level two (**) = M—1 × SD, and level three (***) = M—1.5 × SD, where “M” and “SD” refer to average and standard deviation, respectively.

### 2.3. Extraction and Evaluation of Shikimic Acid from Star Anise by HPLC

In this experiment, 0.4 g of powdered star anise fruit was weighed into 20 mL of 90% methanol. The extraction of the sample was done by sonification (Bransonic, M2800-J, Japan) for 60 min at 30 °C and further diluted to 1 mg/mL. As a standard, synthetic shikimic acid purchased from Tokyo Chemical Industry was also dissolved in 90% methanol at a concentration of 1 mg/mL. Both the crude extract and the synthetic compound were filtrated through a 0.45 μm filter for the HPLC analysis. Triplicate injections of 10 µL to HPLC were performed. The HPLC (PX-8020) was equipped with a degasser, an autosampler (AS-8020). The separation of the samples was performed using TSKgel ODS column (250 mm × 4.6 mm; 5 µm, TOSOH Bioscience, Japan) and 0.1% o-phosphoric acid in water as the mobile phase. The analysis was carried out at a wavelength of 210 nm using a UV-VIS detector (UV-8020).

### 2.4. Bioassay for Plant Extract and Pure Compound

The strength of allelochemicals can be assessed by the biological activity of a compound as expressed by EC_50_. The EC_50_ is the effective concentration of a compound to induce half of the maximum action. This activity is expressed by the specific concentration of the compound and is termed as “specific activity” [15,16]. In this experiment, specific activity (EC_50_) was determined based on the concentration of crude extract or pure compound and the percentage inhibition of plant growth. Compounds with a high specific activity can potentially be used as pesticides. Another term that characterizes allelochemical action is the “total activity”. The total activity of a compound is a function of its specific activity (EC_50_) and its content in the plant and, via this value, the role and influence of a compound on the allelopathic effect can be evaluated [17]. According to Fujii and Hiradate [16], L-3,4-dihydorxyphenylalanine (L-DOPA), isolated from velvet bean (*Mucuna pruriens* (L.) DC. *var. utilis* (Wall. ex Wight) Baker ex Burck), and durantanins from golden dewdrop (*Duranta repens* L.), have a high total activity amounting to 200.

A far more influential compound is 1-o-cis-cinnamoyl-b-d-glucopyranose-6-o- (4-hydroxy-2-methylenebutyroyl)-1-o-cis-cinnamoyl-b-d-glucopyranose, isolated from *Spiraea thunbergii*, which has a very high specific activity, as well as a high total activity (1000) [15]. The seeds of lettuce (*Lactuca sativa* L.) were used as the receptor plant for the bioassay for initial screening. Other weeds and crop species including red clover (*Trifolium pratense* L.), white clover (*Trifolium repens* L.), alfalfa (*Medicago sativa* L.), and bird’s-foot trefoil (*Lotus corniculatus* L.) seeds were also used for the bioassay. The working solutions of star anise with 90% methanol extract were at concentrations of 20, 50, 100, 200, 500, and 1000 ppm. The synthetic shikimic acid was also tested for potential inhibitory effect on the receptor species. Ten milligrams of pure shikimic acid was dissolved in 10 mL of 90% methanol and further diluted to obtain the solutions at concentrations of 20, 50, 100, 200, 500, and 1000 ppm. One milliliter of the different concentrations of plant extract or pure shikimic acid was added to 27 mm filter papers in same size glass Petri dishes and dried completely in vacuo. The control treatments were set up with only 90% methanol without plant extract or pure shikimic acid. After adding distilled water (0.7 mL), five pre-germinated (20 h at 22 °C in the dark) lettuce seeds were placed in each of the dishes with three replications and incubated at 22 °C in darkness for 52 h. The other pre-germinated seeds of white clover, red clover, alfalfa, and bird’s-foot trefoil were also used for the bioassay. Finally, the inhibition of radicle elongation was determined by comparing the radicle lengths of treated plants with that of the control (same as Section 2.2). The effective concentrations required to induce half-maximal inhibition of growth (EC_50_) were calculated according to the linear relationship between concentration and per cent inhibition of plant growth.

## 3. Results and Discussion

### 3.1. Allelopathic Potential by Sandwich Method

**Figure 1:**
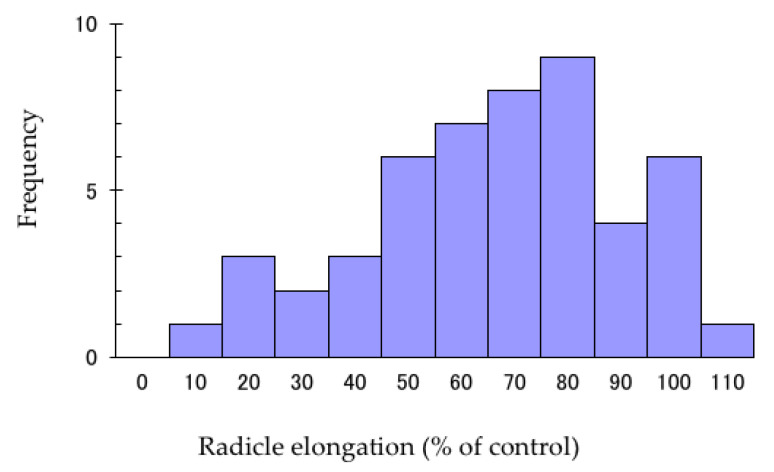
Normal distribution of 50 plant species based on the effects of 10 mg sample treatment on radicle growth of lettuce by sandwich method.

The potential allelopathy of the 50 medicinal plant species was evaluated using the sandwich method. The lettuce radicle and hypocotyl elongation percentages based on the effects of leachates from the oven-dried samples of 50 medicinal plants are shown in Table 1. The results of lettuce radicle elongation for 10 mg sample treatment conformed to the normal distribution (Figure 1). The allelopathic effect on lettuce radicle elongations varied among the different plant species evaluated. Some plant species showed inhibitory effects on lettuce growth and others showed stimulatory effects on lettuce growth. The radicle and hypocotyl elongations of lettuce seedlings, when treated with 10 mg of the samples, were in the range of 14–110% and 25–135% of the control. The lowest radicle elongation (14% of control) was caused by the fruits of *Illicium verum.* Further to this, we observed radicle growth of 20–40% in 5 species, 41–60% in 9 species, 61–80% in 15 species, and 81–100% in 12 species. For the evaluation of the allelopathic activity, the concept of the “standard deviation value” (SDV) was adopted in this study [14]. The mean and standard deviation were calculated, and the criterion of the SDV was evaluated. According to the result of the sandwich method, leaf leachates of five plant species showed strong inhibitory activity. These species were *Chenopodium glaucum* L. (33%), *Nitraria tangutorum* Bobr. (27%), *Stachys geobombycis* C. Y. Wu. (22%), *Gossypium herbaceum* L. (20%), and *Illicium verum* Hook. f. (14%).

On the contrary, seven species showed growth stimulatory activity (radicle elongation > 100% of control) on lettuce. This indicated that the interaction between plants not only include inhibitory effects, but also stimulatory effects. The species that caused stimulatory activity on the growth on lettuce were *Senecio scandens* (101%), *Amaranthus viridis* (102%)*, Arnebia euchroma* (104%)*, Sinapis alba* (106%)*, Armeniaca vulgaris* (107%)*, Carya cathayensis* (108%), and *Morus macroura* (110%). The phenomenon of growth stimulation by plant leachates has been reported in previous studies [9,12,13,14].

In this study, the fruits of *Illicium verum* Hook. f. showed the highest inhibitory activity on growth elongation (14% of lettuce radicle growth). Moreover, the allelopathy activity of *Illicium verum* Hook. F. fruit has not been widely reported. *Illicium verum* Hook. f. (star anise) belongs to the *Illicium* genus, and the highest percentage of shikimic acid was discovered in this genus. Avula et al. [18] reported comparative data from *Illicium* species in which shikimic acid content ranged from 3.56% to 24.8% and levels of shikimic acid in *Illicium* species were higher than in most other plants. Star anise is native to southern China and northern Vietnam and is grown almost exclusively in southern China. *I. verum* has also been reported to possess antimicrobial [19] and antioxidative properties [20], as well as significant anticancer potential [21]. However, the allelochemical involved in the growth inhibitory activity of the leachates of star anise fruits is not reported.

### 3.2. HPLC Analysis of Shikimic Acid in Star Anise

**Figure 2:**
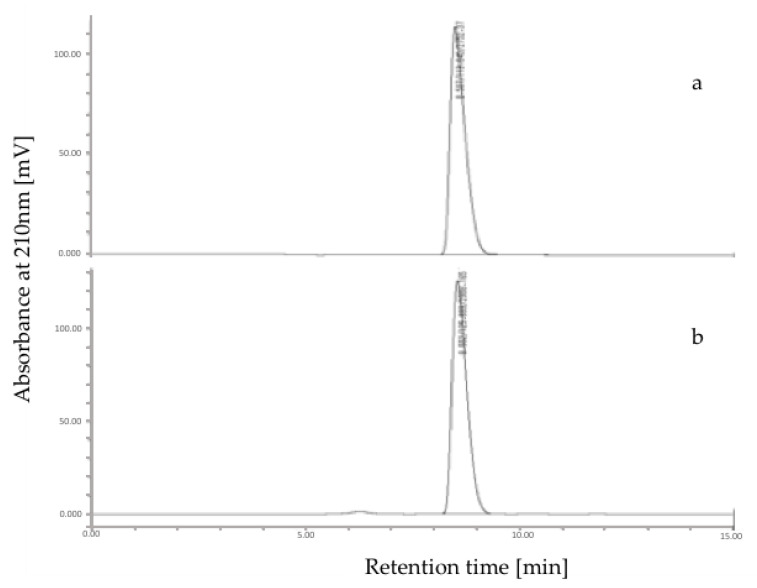
The HPLC chromatogram of standard shikimic acid (**a**) and *I. verum* crude extract (**b**).

Identification and quantification of shikimic acid in the fruit of star anise were conducted by using reversed-phase High-Performance Liquid Chromatography (HPLC). The data were evaluated according to the retention time and UV spectrum of the standard compound. The quantification of shikimic acid was obtained from the calibration equation from the peak areas of the standard solutions at the different concentrations. The HPLC chromatograms of standard shikimic acid and star anise are shown in Figure 2. According to the calibration equation, shikimic acid contents of star anise were determined. It was found that the fruits of star anise contained relatively high shikimic acid content (7.1% dry weight).

### 3.3. Phytotoxicity of Star Anise Crude Extract and Authentic Shikimic Acid

**Figure 3:**
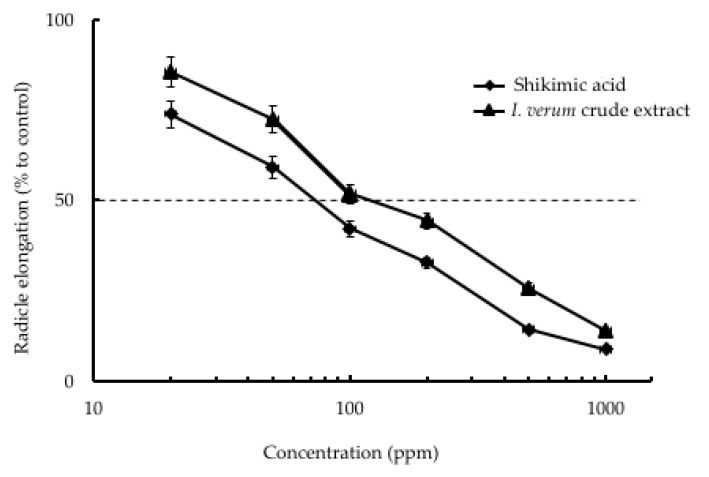
Effect of shikimic acid and *I. verum* crude extract on the radicle elongation of lettuce seedlings. The data are the mean ± standard deviation (*n* = 3).

**Figure 4:**
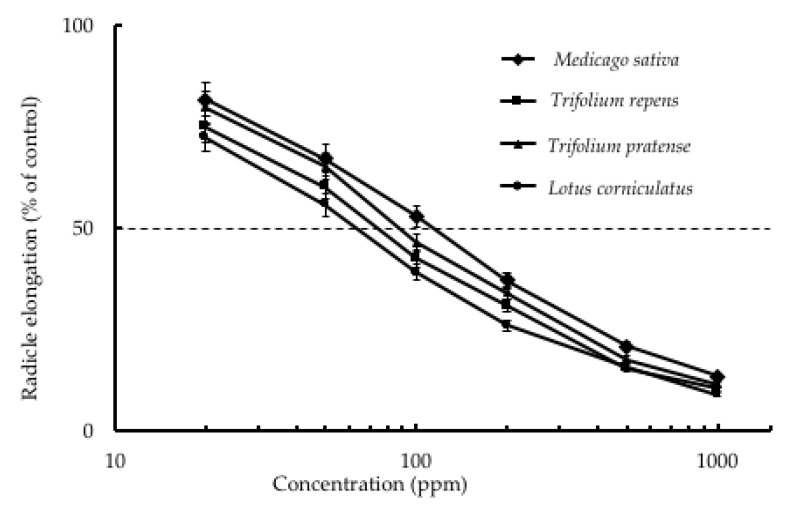
Effect of methanol crude extract of *I. verum* fruit on the radicle elongation of test species. The data are the mean ± standard deviation (*n* = 3).

In this experiment, the specific activity (EC_50_) was determined based on the concentration of crude extract or pure compound and per cent inhibition of plant growth elongation. The fruit of star anise was chosen as the candidate species since it showed the highest growth inhibitory effect on lettuce radicle elongation. The seeds of lettuce (*Lactuca sativa* L.) were used for the bioassay test plant. Other weed and crop species including red clover, white clover, alfalfa, and bird’s-foot trefoil seeds were also used in this bioassay. Shikimic acid has been reported to be the main compound in the fruit of star anise. Liu et al. [22] reported significant variation among samples from the different areas, with the shikimic acid content ranging from 2.2% to 15% in Chinese star anise. Shikimic acid is an intermediate of the shikimic acid pathway [23], which is involved in the synthesis of aromatic metabolites in plants and micro-organisms [24,25,26,27]. In addition, shikimic acid is a phenolic acid that is known to possess several biological activities. Phenolic acids such as *p*-hydroxybenzoic, vanillic, *p*-coumaric, and syringic, and ferulic acids are the main category of allelochemicals. These phenolic acids have been identified as allelopathic agents in natural and agroecosystems [28,29]. However, we focused on the evaluation of inhibitory activity of star anise and shikimic acid. As shown in Figure 3, both the extracts of star anise and pure shikimic acid showed strong inhibitory effects on lettuce growth. The EC_50_ of shikimic acid and the crude extracts of star anise fruit on lettuce radicle were 80 and 100 ppm, respectively. Both showed strong inhibitory activities, and it was observed that shikimic acid was the main compound in star anise with a relatively high content of 7.10% regarding the HPLC analysis. The results indicated that radicle growth inhibition of lettuce caused by the crude extract of star anise fruit could be explained by the contribution of the biological activity of shikimic acid (Figure 3). However, the contributions of other compounds cannot be neglected. Shikimic acid also inhibited the growth of other test plants including *Trifolium pratense* L., *Trifolium repens* L., *Medicago sativa* L., and *Lotus corniculatus* L. Shikimic acid exhibited the highest inhibitory effect on the growth of bird’s-foot trefoil with a specific activity (EC_50_) of 60 ppm, followed by white clover (70 ppm), red clover (90 ppm), and alfalfa (100 ppm), in that order (Figure 4).

## 4. Conclusions

This study focused on Chinese medicinal plants and the evaluation of their allelopathic activity. The results of the study indicated that among the 50 evaluated medicinal plant species from China, the fruits of *Illicium verum* Hook. f. (star anise) showed the highest allelopathic activity. Chinese star anise is the main source of shikimic acid. In this study, HPLC analysis demonstrated that the shikimic acid content in Chinese star anise was 7.10%. The results of the phytotoxic test for synthetic shikimic acid and *I. verum* crude extract showed that the radicle growth inhibition of lettuce caused by crude extract of star anise could be explained by the contribution of the biological activity of shikimic acid. For these reasons, shikimic acid has great potential in allelopathy and eco-friendly agriculture.

## Figures and Tables

**Table 1:** Radicle and hypocotyl elongation of lettuce on 50 Chinese medicinal plants tested by sandwich method.

Scientific Name	Plant Parts	Elongation (% of control)				Criteria *
		10 mg		50 mg		
		R%	H%	R%	H%	
*Illicium verum* Hook. f.	fruit	14.0	25.3	5.3	13.3	***
*Gossypium herbaceum* L.	root	20.3	53.0	9.3	27.0	***
*Stachys geobombycis* C. Y. Wu	root	22.0	76.3	11.7	49.3	***
*Nitraria tangutorum* Bobr.	leaf	26.3	71.3	13.3	41.0	***
*Chenopodium glaucum* L.	leaf	33.0	93.3	9.3	35.0	***
*Capsicum annuum* L.	fruit	35.3	74.0	11.0	27.3	**
*Lycium ruthenicum* Murray	fruit	41.7	66.3	15.0	50.3	**
*Physalis alkekengi* L.	fruit	43.3	90.0	18.3	25.0	**
*Indigofera tinctoria* L.	leaf	45.0	90.0	29.3	104.0	**
*Salix sinopurpurea* C. Wang et C. Y. Yang	leaf	52.3	78.0	34.3	61.0	*
*Araliachinensis* L. var. *Dasyphylloides* Hand. Mazz.	root	54.0	67.3	24.0	26.3	*
*Glycyrrhiza uralensis* Fisch.	root	56.0	104.3	28.0	62.3	*
*Wrightia laevis* Hook. f.	leaf	56.7	97.3	21.0	79.0	*
*Lycium dasystemum* Pojarkova	fruit	58.3	77.0	37.3	61.3	*
*Symplocos dolichotricha* Merr.	root	59.0	87.3	25.0	60.3	
*Phtheirospermum japonicum* Kanitz	leaf	60.0	122.0	28.3	87.0	
*Leonurus artemisia* (Lour.) S. Y. Hu	leaf	61.3	109.0	38.0	101.3	
*Saussurea involucrata* Sch.-Bip.	fruit	62.0	125.3	30.0	84.3	
*Corydalis bungeana* Turcz.	leaf	64.0	116.3	23.3	67.0	
*Gynostemma laxum* (Wall.) Cogn.	leaf	65.3	114.0	27.0	65.3	
*Carthamus tinctorius* L.	fruit	67.0	122.3	23.3	76.0	
*Cynomorium songaricum* Rupr.	root	68.0	130.0	27.3	61.0	
*Agastache rugosa* O. Ktze.	fruit	70.3	83.0	31.7	39.3	
*Impatiens balsamina* L.	fruit	71.3	134.0	22.3	82.0	
*Edgeworthia chrysantha* Lindl.	fruit	72.7	86.3	40.3	91.0	
*Cistanche deserticola* Ma	root	74.3	110.0	90.3	106.0	
*Atractylodes lancea* (Thunb.) DC.	root	75.0	99.3	45.0	80.3	
*Amygdalus communis* L.	fruit	77.0	98.0	50.3	74.0	
*Trigonella foenum-graecum* L.	fruit	78.3	107.0	44.7	78.3	
*Phyllanthus emblica* L.	fruit	78.7	126.0	39.3	85.0	
*Ziziphus jujuba* Mill.	fruit	80.3	98.0	51.7	70.0	
*Adenophora stricta* Miq.	fruit	81.3	106.3	45.0	81.7	
*Cicer arietinum* L.	fruit	81.0	126.0	45.3	88.0	
*Punica granatum* L.	fruit	84.3	111.3	25.0	65.7	
*Paeonia lactiflora* Pall.	fruit	85.0	99.0	41.3	94.0	
*Spenceria ramalana* Trimen	root	87.0	111.0	49.0	67.3	
*Adenia chevalieri* Gagnep.	fruit	88.3	107.3	18.0	46.0	
*Fritillaria walujewii* Regel	root	88.7	104.0	42.0	53.3	
*Heracleum scabridum* Franch.	root	89.0	127.3	28.0	64.3	
*ulipa iliensis* Regel	fruit	90.3	100.0	60.3	72.0	
*Dendrobium nobile* Lindl.	fruit	92.0	111.3	68.0	104.3	
*Equisetum hyemale* L.	fruit	93.7	138.0	61.3	100.0	
*Munronia sinica* Diels	root	96.0	120.3	31.0	53.3	
*Senecio scandens* Buch. -Ham. ex D. Don	fruit	101.3	152.0	47.3	104.0	
*Amaranthus viridis* L.	leaf	102.3	139.3	55.0	93.0	
*Arnebia euchroma* (Royle) Johnst.	leaf	104.3	109.0	90.0	106.3	
*Sinapis alba* L.	fruit	106.0	111.3	86.3	108.0	
*Armeniaca vulgaris* Lam.	leaf	107.0	118.0	65.3	86.3	
*Carya cathayensis* Sarg.	leaf	108.7	116.3	65.0	94.0	
*Morus macroura* Miq.	leaf	110.0	135.3	72.0	82.3	
M—0.5 × SD		58.7	91.5	27.6	58.5	
M—SD		46.4	79.4	17.0	46.1	
M—1.5S × D		34.1	67.3	6.5	33.8	

R% = Radicle elongation percentage, H% = Hypocotyl elongation percentage. [*] indicates the plant growth inhibition compared with the radicle elongation of the control (*L. sativa*) using the standard deviation variance (SDV), where: * = M—0.5 × SD, ** = M—1 × SD, and *** = M—1.5 × SD. Each [*] indicates a stronger level of growth inhibition. M = Mean of radicle elongation, SD = Standard deviation of control radicle elongation.
